# A marker-less human motion analysis system for motion-based biomarker identification and quantification in knee disorders

**DOI:** 10.3389/fdgth.2024.1324511

**Published:** 2024-01-23

**Authors:** Kai Armstrong, Lei Zhang, Yan Wen, Alexander P. Willmott, Paul Lee, Xujiong Ye

**Affiliations:** ^1^ Laboratory of Vision Engineering, School of Computer Science, University of Lincoln, Lincoln, United Kingdom; ^2^ School of Sport and Exercise Science, University of Lincoln, Lincoln, United Kingdom; ^3^ MSK Doctors, Sleaford, United Kingdom

**Keywords:** biomarkers, biomechanics, machine learning, human pose estimation, human mesh recovery, patient monitoring, automated rehabilitation

## Abstract

In recent years the healthcare industry has had increased difficulty seeing all low-risk patients, including but not limited to suspected osteoarthritis (OA) patients. To help address the increased waiting lists and shortages of staff, we propose a novel method of automated biomarker identification and quantification for the monitoring of treatment or disease progression through the analysis of clinical motion data captured from a standard RGB video camera. The proposed method allows for the measurement of biomechanics information and analysis of their clinical significance, in both a cheap and sensitive alternative to the traditional motion capture techniques. These methods and results validate the capabilities of standard RGB cameras in clinical environments to capture clinically relevant motion data. Our method focuses on generating 3D human shape and pose from 2D video data via adversarial training in a deep neural network with a self-attention mechanism to encode both spatial and temporal information. Biomarker identification using Principal Component Analysis (PCA) allows the production of representative features from motion data and uses these to generate a clinical report automatically. These new biomarkers can then be used to assess the success of treatment and track the progress of rehabilitation or to monitor the progression of the disease. These methods have been validated with a small clinical study, by administering a local anaesthetic to a small population with knee pain, this allows these new representative biomarkers to be validated as statistically significant (p-value <0.05). These significant biomarkers include the cumulative acceleration of elbow flexion/extension in a sit-to-stand, as well as the smoothness of the knee and elbow flexion/extension in both a squat and sit-to-stand.

## 1 Introduction

**Figure 1:**
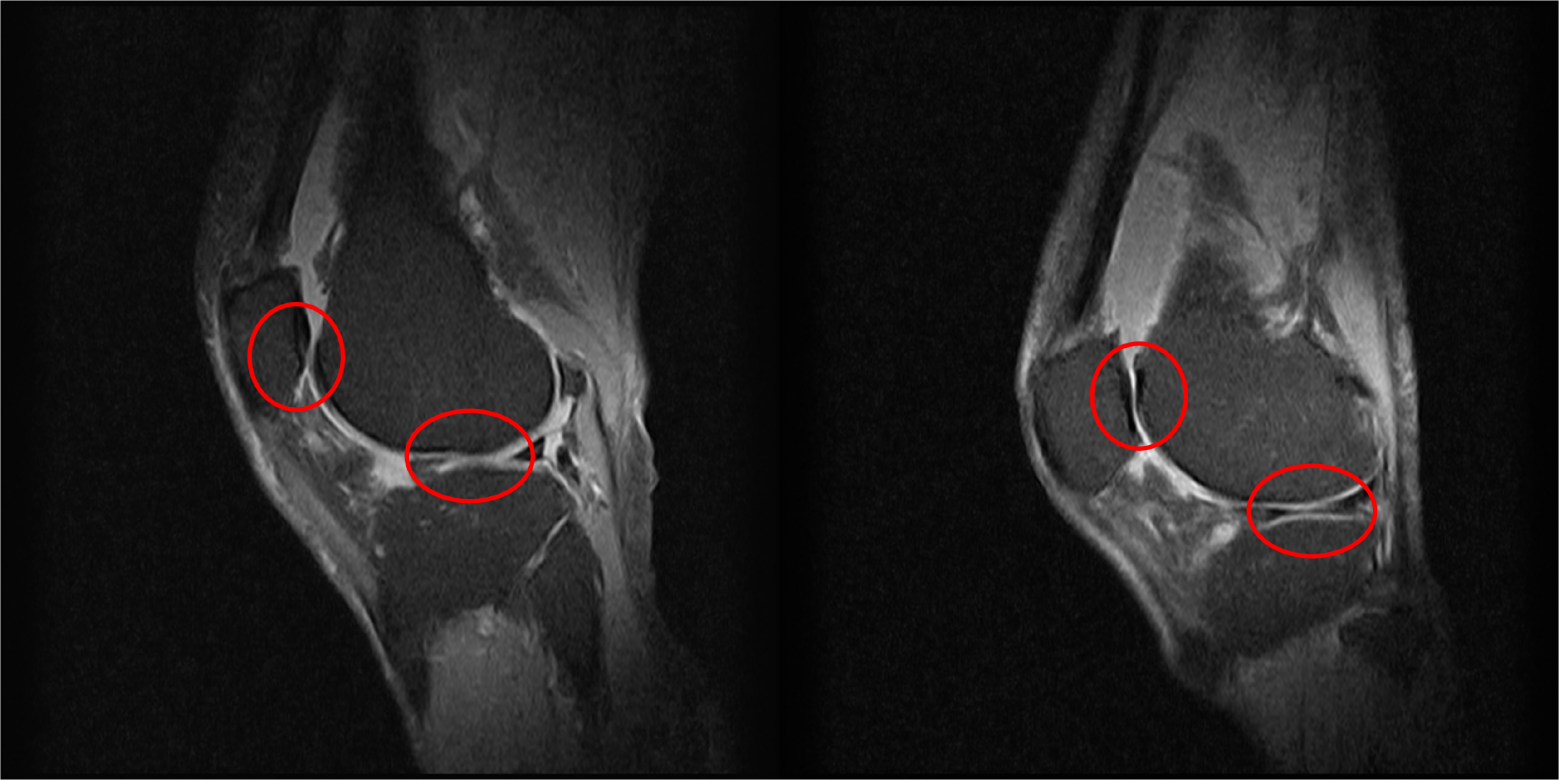
Two examples of knee MRIs in the sagittal view from this study’s patients to highlight the severity of osteoarthritis, as shown by the lack of cartilage around the knee joint.

The knee, a remarkable yet vulnerable joint, stands as one of the most frequently afflicted areas in the human body. Amid its susceptibility to injuries, knee osteoarthritis (OA) emerges as the most prevalent joint disorder in the United States, affecting a substantial portion of the population (1). This widespread prevalence underscores the critical need for effective diagnostic and intervention strategies in the realm of knee health. Factors such as age, weight, and occupation contribute to the risk of developing knee OA (2). In the United Kingdom, the burden of knee OA is evidenced by over 90,000 total knee replacements annually, a testament to the impact on individuals’ daily lives and the strain on healthcare systems (3, 4). As these interventions come at a considerable cost, surpassing £7,000 on average per procedure or a cost per Quality-adjusted Life Year (QALY) gained exceeding £1,300, the economic implications are significant, with the UK’s National Health Service (NHS) expending over £600 million annually on knee-related procedures (5). Traditionally, osteoarthritis has been diagnosed with magnetic resonance imaging as shown in Figure 1. This brings economic and healthcare burdens and necessitates innovative approaches to both diagnosis and treatment, paving the way for advancements in medical imaging and motion analysis techniques.

Expanding on the diagnostic landscape, recent developments in marker-based motion capture (MoCap) have provided valuable insights into the biomechanics of the knee and its relationship to osteoarthritis. Utilising retroreflective markers, marker-based MoCap enables precise tracking of joint movements, allowing for a detailed analysis of gait patterns, joint kinematics, and overall knee function (6–8). Studies by Sparkes et al. and Duffell et al. have demonstrated the efficacy of gait analysis in distinguishing individuals with knee osteoarthritis from healthy counterparts, highlighting the potential of MoCap as a diagnostic tool (6, 7). Moreover, Robbins et al. delved into risk factors associated with both non-traumatic and post-traumatic knee osteoarthritis, showcasing the versatility of marker-based MoCap in understanding disease progression (8).

While marker-based MoCap provides valuable data, its adoption in clinical settings presents challenges, including the need for highly trained experts, time-consuming data collection, and the demand for dedicated laboratory spaces (9–11). Despite these limitations, marker-based MoCap remains a cornerstone in biomechanics research, offering unparalleled insights into knee function and pathology. Recent strides in technology have furthered the field with a promising era of marker-less motion capture. Fueled by advancements in human pose estimation techniques, this innovative approach provides a compelling alternative for the analysis of human movement, particularly in clinical settings (12). Unlike marker-based methods that rely on the placement and tracking of retroreflective markers, marker-less motion capture extracts intricate details of human pose directly from standard RGB images and videos (13). This transition from marker-based to marker-less motion capture introduces new possibilities while addressing some of the challenges associated with traditional marker-based approaches.

Marker-less motion capture, while helping alleviate some of these problems, serves as an effective tool in the analysis of human pose, particularly in uncontrolled environments (12). However, applying these techniques in a clinical setting requires a reproducible result in any setting. The primary challenge lies in the difficulty of controlling the clinical environment. Many clinical locations have natural lighting whose directions vary throughout the day, potentially leading to incorrect human motion sequences (14). Additionally, variations in patient attire and potential occlusion of body parts due to clothing further complicate marker-less motion capture and biomechanics analysis (15–17). These conditions must be meticulously controlled to minimise data variation. Moreover, each patient has different functional capabilities, necessitating careful selection of actions to develop a series of tests that everyone can perform.

In response to these challenges, this study aims to introduce an automated system designed for the analysis of clinical motion data captured from a standard 2D RGB video camera. This system aims not only to quantify, identify, and measure objective diagnostic biomarkers but also to present a faster and more cost-effective alternative to the current gold standard of marker-based MoCap. To address the complexities and variabilities inherent in clinical data capture environments, our approach involves the development of a fully end-to-end marker-less biomechanics solution. We acknowledge the intricate nature of clinical tests, the variability in data capture environments, and the complexity of result analysis. To tackle these hurdles, we propose a simple yet robust protocol and framework designed to function in any environment. By adhering to a standardised protocol endorsed by medical professionals, our approach not only reduces variability in data but also ensures clinical relevance throughout the biomechanics analysis process

This approach utilises human pose estimation, a process of locating the positions of human joints from images and videos (18). The technique, owing to its portability, low cost, and accessibility, has gained prominence in the fields of sports science and clinical biomechanics analysis (19). Recent advancements in human pose estimation have facilitated rapid and accurate marker-less MoCap using standard RGB images and videos (13). These innovations include extracting a 3D mesh of a person from the 2D RGB image, enhancing anatomical detail for face, body, and hand features. Notably, the current standards for the 3D mesh model, such as SMPL (Skinned Multi Person Linear) and SMPL-X, undergo continuous improvement and iterations depending on the desired use (20). When applied to videos, these SMPL models utilise a motion discriminator generative adversarial network (GAN), enabling a model that accurately represents human motion with assistance from the temporal domain (21, 22).

While the aforementioned advancements in human pose estimation and marker-less motion capture have significantly enhanced our ability to capture intricate details of human motion, their application extends beyond sports science and general biomechanics. In particular, these technologies lay the foundation for a transformative approach in clinical settings, where the need for precise motion analysis holds significant implications. However, despite the strides made in accurately capturing human pose data, the clinical significance of such data for specific conditions, such as knee disorders, has been an under-explored territory (23, 24).

Building on this gap in research, our methodology takes a crucial step in addressing the need for a comprehensive analysis of clinical motion data. By specifically tailoring our approach to assess motion data before and after treatment, we not only contribute to the development of effective intervention strategies but also provide a means for tracking rehabilitation progress and evaluating the progression of knee disorders. The integration of advanced human pose estimation and marker-less motion capture techniques, as demonstrated in the preceding paragraph, forms the cornerstone of our sophisticated and clinically relevant approach to capturing and interpreting human motion data.

In pursuit of these objectives, this study seeks to assuage concerns surrounding current methods employed in clinical environments. Our proposed approach leverages state-of-the-art marker-less motion capture systems combined with kinematics analysis, aiming to identify biomarkers and establish a robust framework for tracking disease progression or rehabilitation progress. Crafted to meet the unique demands of clinical settings—fast, accessible, cost-effective, and portable—our method incorporates manual feature calculation to provide explainable results to both patients and clinicians. Further enhancing interpretability, we employ Principal Component Analysis (PCA) to extract those features that have greatest power to discriminate between different conditions. The output from our proposed pipeline culminates in a medical report tailored for presentation to both clinicians and patients. To demonstrate the efficacy of our approach, we conclude with a small clinical case study, administering a local anaesthetic to a population with knee pain, resulting in the identification of novel motion-based biomarkers that are not only generalisable but also action-specific.

## 2 Materials and method

**Figure 2:**
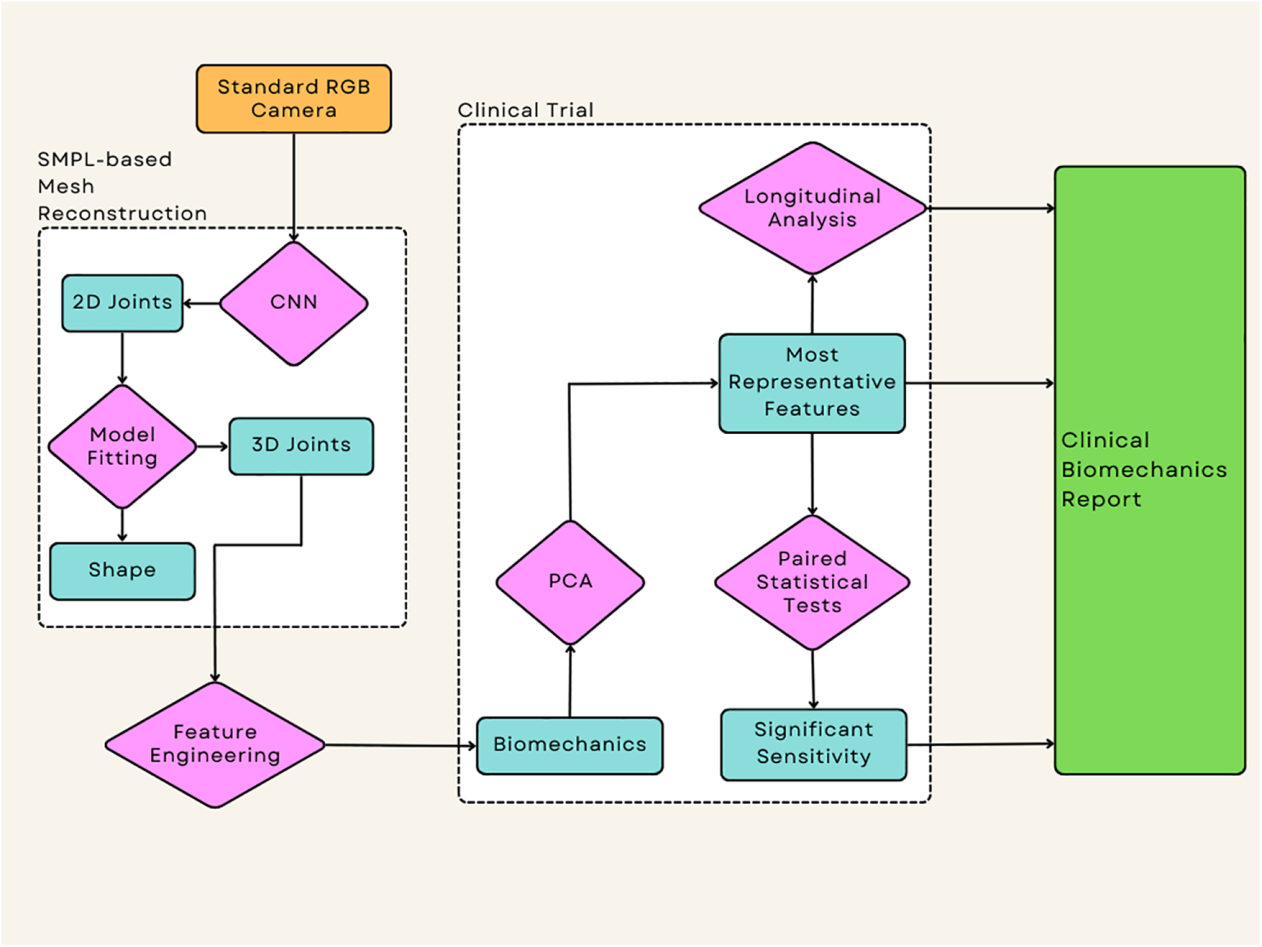
This represents the flow of the data from the source of the videos to the output of the statistical tests which allow the extraction of any significant data, this application has been applied to a clinical case study to examine the effectiveness of each technique when applied to intervention success.

The flow of data from collection to the extraction of clinically relevant and statistically significant biomechanics features is outlined in Figure 2. The process begins with 1080p standard videos being recorded on an Azure Kinect RGB-D camera at 30 frames per second. The camera height was 1.2m and was placed at a distance of 3m away from the subject. Each video was recorded with the participant facing the camera, this reduces the effects of occluded joints on the motion capture technique. The participants performed at least 3 repeats each of a sit-to-stand and squat action, this protocol has been designed by clinicians to use simple actions that all participants can perform. Simultaneously, the diagnostic efficacy of the sit-to-stand and squat actions are substantiated by existing literature, thereby fortifying its diagnostic power (25–27). For the squat, the participants were asked to squat as low as they could and then immediately return to a standing position. For the sit-to-stand; the participants were asked to stand up from a chair, use of arms was permitted out of safety concerns, then returned to a seated position.

Inference of videos was performed using an Nvidia GTX 1080ti using a pre-trained checkpoint, where the average inference time is 40 seconds per 10 seconds of video.

### 2.1 Clinical case study

To show the sensitivity of these methods in a clinical environment these techniques were performed on a small case study of 20 participants. The demographic for the study was kept broad to account for a variety of situations, the selection criteria were men and women over the age of 55 and a diagnosis of knee pain. It is important to note that it is almost impossible to isolate this to pain in a single limb, therefore, patients with bilateral knee pain were also recruited to the study as long as one side was found to be worse than the other. As age is a well-known risk factor for OA this increases the chance that their diagnosis is due to knee OA rather than an injury (28).

Each participant received a local anaesthetic injected into the knee with the diagnosed pain. This removes the psychological change to movement caused by pain, providing the biomechanics analysis with a clear before and after treatment and allowing us to assess the sensitivity of each capture method.

The study protocol was approved by the University of Lincoln Ethics, Governance & Regulatory Compliance Committee, the study was performed following relevant institutional guidelines and regulations. All participants provided written informed consent before any data collection.

### 2.2 Human mesh recovery

The SMPL-X model used in this study consists of a total of 10,475 mesh vertices surrounding a skeleton of 54 joint locations. The model is defined by the function $M(\theta ,\beta ,\psi )\;:\;R^{\left|\theta |\times |\beta |\times |\psi \right|}\to R^{3}N$, where θ represents the body pose, ψ represents the facial parameters, β represents the face and hand shape parameters, and N represents the denotes the number of frames in the video (20). This body model allows for inferences to be made from standard RGB images or image sequences, accounting for not only the pose of a subject but also the shape, providing more detail than a standard human pose estimation technique such as OpenPose or BlazePose (13, 29).

The standard RGB videos were fed into a mesh reconstruction pipeline based on the Video Inference for the Human Body Pose and Shape Estimation (VIBE) model, which predicts the SMPL parameters of a given participant based on monocular RGB video. The adoption of the VIBE model in our experiment was due to its ability to encode both spatial and temporal cues into the data using adversarial training in a deep neural network with a self-attention mechanism (21). To ensure reproducibility and optimal accuracy, the training and implementation details were implemented using the parameters described by Kocabas et al. (21), including sequence length=16, temporal encoder=2-layer GRU with a hidden size of 1,024 and learning rate of $5\times 10^{-5}$ and an Adam optimiser, SMPL regressor=2 fully connected layers of size 1,024, motion discriminator=2-layer GRU with a hidden size of 1,024 and a learning rate of $1\times 10^{-4}$, and the self attention=2 MLP layers of size 1,024 with *tanh* activation. The model was trained using InstaVariety (30) as the 2D ground-truth dataset, MPI-INF-3D (31) as the ground-truth 3D dataset, and 3DPW as the 3D ground-truth dataset for evaluation purposes; this training consisted of 30 epochs with 500 iterations per epoch and a batch size of 32 (32).

### 2.3 Whole body kinematic feature extraction

**Figure 3:**
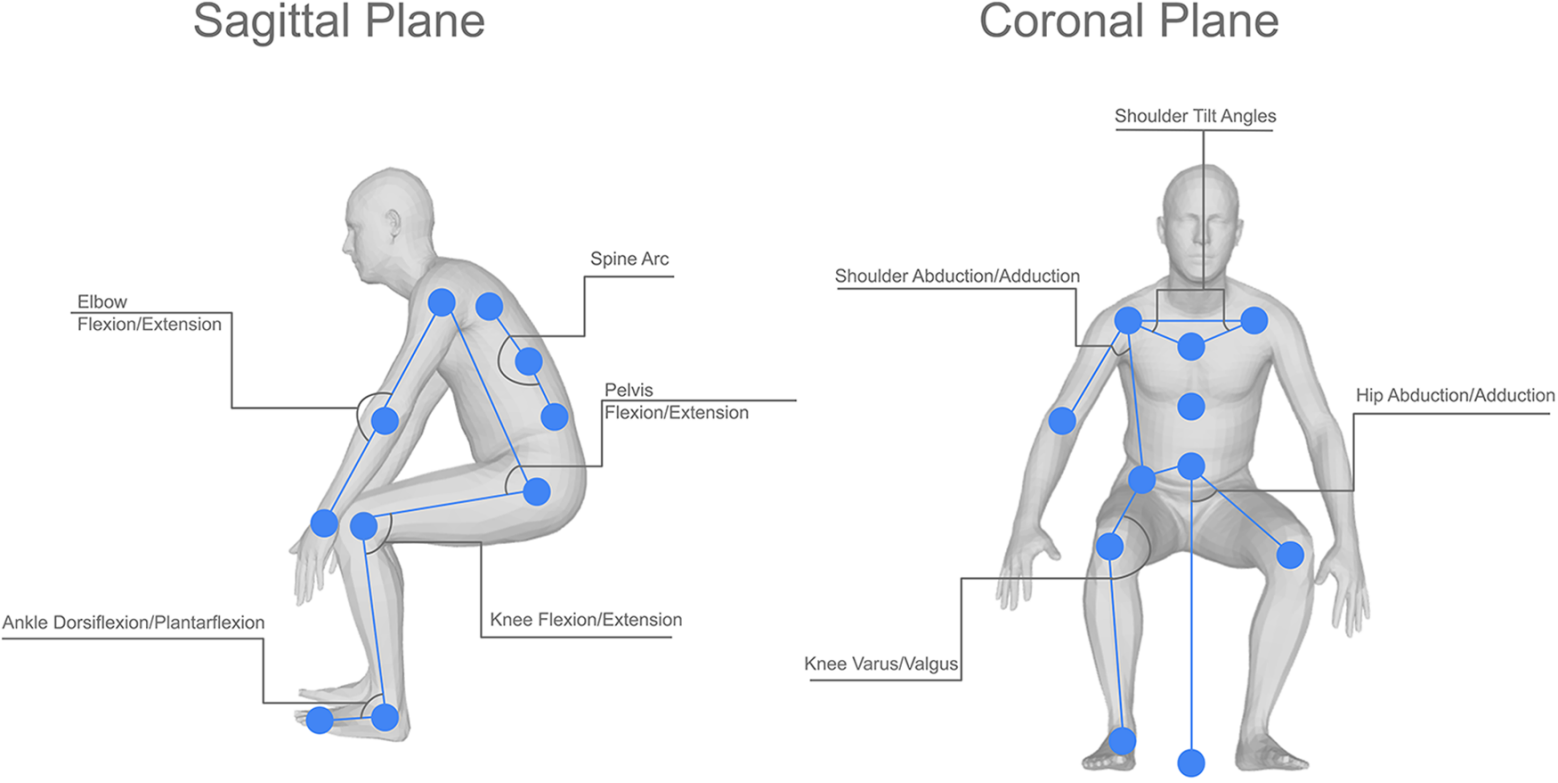
Visual representation of the joint angles used in this study, showing both the sagittal and coronal joint angles and the location of the joint centres used in the calculations for each angle.

A visual representation of the joint angles used can be seen in Figure 3, this shows the joint angles selected in both the sagittal and coronal planes. Each of the joint angles, *θ*, was calculated using a base formula as shown in Equation 1, where k, h, and a represent the joint centres in a given plane of motion. These joint angles were then calculated at every frame in the video to produce joint angle sequences. This was then performed for each of the participant’s repeats, this allows the extrapolation of a mean, minimum, and maximum curve by comparing the kinematics at the same frame in each repeat. The decision was also made not to time normalise the data under the assumption that any extreme changes to the length of an action could be caused by the participant’s clinical pathology.

For vectors:m=h−k, n=a−k, and p=h−a

(1) $$\theta ={\text{cos}}^{-1}\left(\frac{|m{|}^{2}+|n{|}^{2}-|p{|}^{2}}{2|m||n|}\right)$$

where k, h, and a, are the positions of the joint centres of any three given joints for example knee, hip, and ankle respectively can be used to calculate the knee angle and ∣.∣ denotes the Euclidean distance between two points.

(2) $$\sigma_{m}=\sqrt{\frac{\sum_{i=1}^{n}(m_{i}-\bar{m}{)}^{2}}{n-1}}$$

The smoothness of the mean, maximum, and minimum curves can then be calculated from the standard deviation of the gradient of a curve, as shown in Equation 2 where n is the number of data points, $m_{i}$ is the gradient of a slope at the $i^{th}$ point calculated as $m_{i}=\frac{\Delta y_{i}}{\Delta x_{i}}$ and $\bar{m}$ is the mean of the gradients. This smoothness value shows how much the slope of a curve varies from point to point, whereby a smoothness closer to 1 identifies a smoother curve. The rotational velocity and acceleration of the knee were calculated from the changes in knee angle over time (Equation 3) A simplified measure to represent the kinematics of the movement was defined as follows: the cumulative absolute rotational acceleration, *J*, was calculated as a representative measure of the explosiveness of the movement or the overall abruptness of the changes in angular velocity (Equation 4).

The rotational velocity ω and acceleration α were calculated from the changes in the knee angle over time, t:

(3) $$\begin{array}{cc} \omega & =\frac{\Delta \theta }{\Delta t}\\ \alpha & =\frac{\Delta \omega }{\Delta t} \end{array}$$

*J* is the cumulative absolute rotational acceleration of an action at a single joint, ∣α∣ is the absolute instantaneous rotational acceleration and *dt* is the change in time.

(4) J=∫∣α∣⋅dt

After the mean, maximum, and minimum joint angles had been prepared for each participant’s squat and sit-to-stand actions, this was fed into the PCA feature extraction component. This method of dimensionality reduction is used to rank the feature importance for each action, allowing only the most important features to be used in the subsequent methods (33). This method used a two-component PCA with single value decomposition, thus creating a linear dimensionality reduction and accounting for both the actions used. As this was performed on each participant, this allows the identification of features that are representative of all participants in the trial. To achieve this the features are ranked by importance in each action, then we analyse the frequency of these features appearing in the ten highest feature importance scores. This allows us to create a histogram to identify which features will be most representative of the whole participant group, as a higher frequency is due to this feature being ranked highly across most participants.

### 2.4 Statistical testing

Two-tailed paired t-tests were performed between the pre- and post-injection values for the cumulative acceleration and smoothness values at the joints identified by PCA as being most important in discriminating between the two conditions. A significance level of 0.05 was used. The pre- and post-injection conditions were compared for both the squat and sit-to-stand movements. In addition to the paired t-tests, Bland-Altman plots, with a range of limits of agreement of 1.96 and approximate confidence intervals as described by Bland and Altman (34), were created to assess the agreement between the pre- and post-injection cumulative acceleration and smoothness for the same selection of joints. For each pair of pre/post-injection observations, the difference between the scores was plotted against the mean of the two scores; the overall plot provides information about the level of variation and whether or not there was any systematic bias.

## 3 Results

### 3.1 Feature engineering and biomarker identification

**Figure 4:**
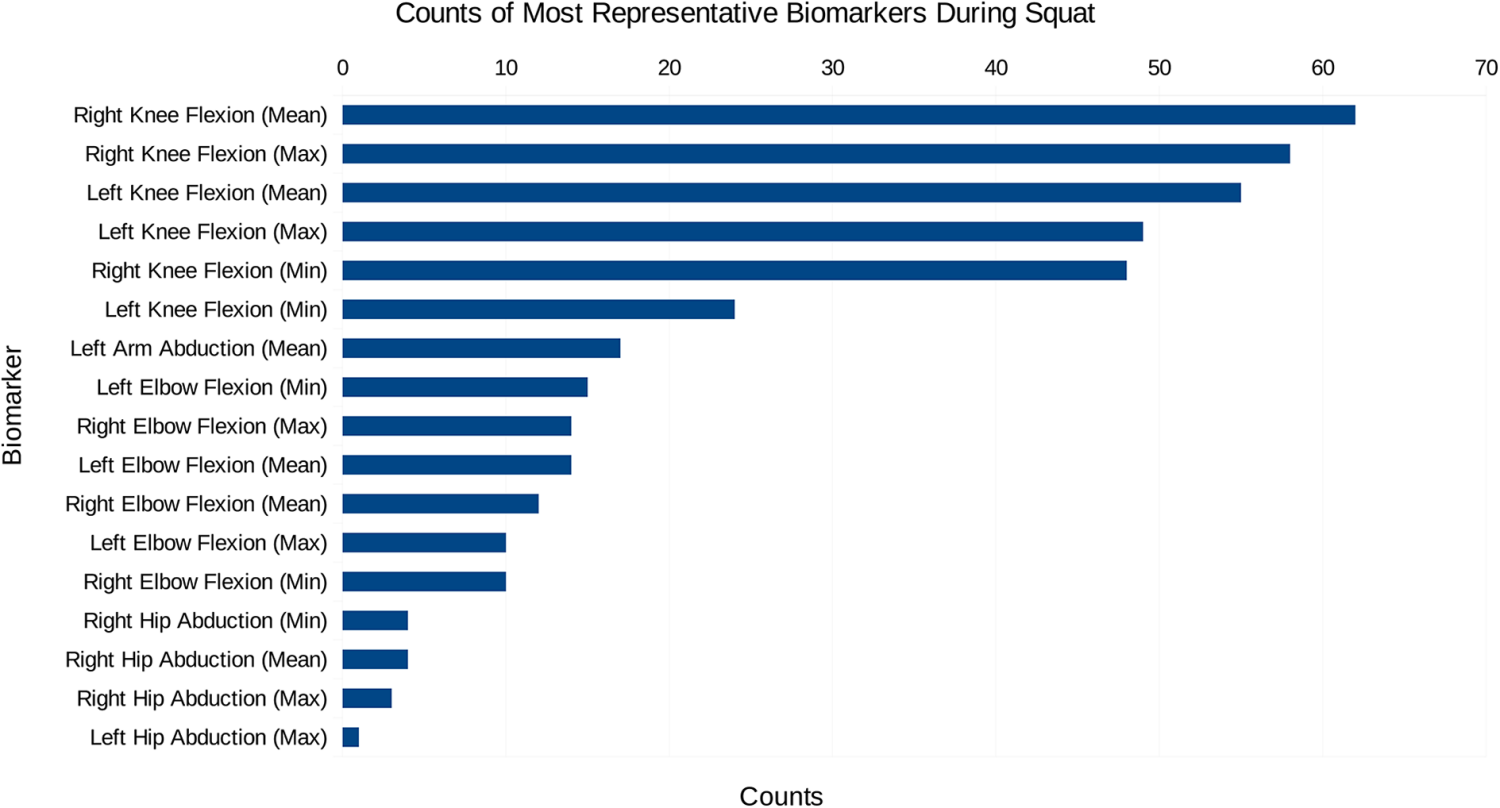
The most common features among the most representative biomarkers during the squat action.

**Figure 5:**
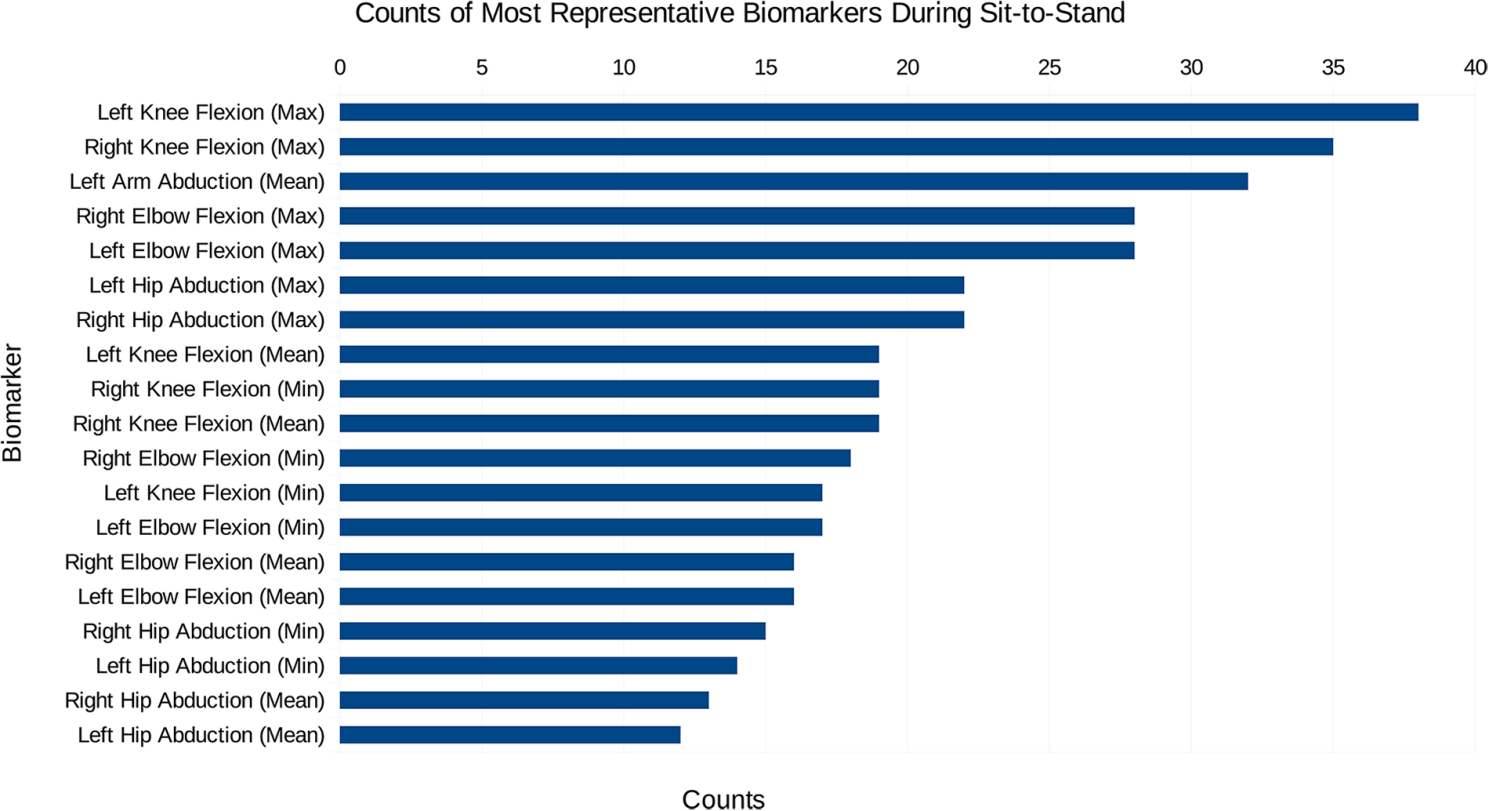
The most common features among the most representative biomarkers during the sit-to-stand action.

Initially, the data created consisted solely of positions and orientations of joints in a 3D Cartesian coordinate system for both a squat and a sit-to-stand action, which first needs to be transformed and engineered into clinically relevant features, such as the knee flexion/extension, hip abduction/adduction, and the ankle flexion/extension. The dimensionality of this data was then reduced and separated into the squat and sit-to-stand actions using the PCA. Performing this PCA for each subject’s actions finds the most representative features as a histogram shown in Figures 4, 5, this was derived from the total counts of each of the top five most represented features of each participant and in each action performed. Figure 4 for example, shows the most represented features among all patients in the squat action to be the mean and maximum knee flexion for both the left and right side. On the other hand, the sit-to-stand feature histogram as shown in Figure 5 shows the most representative features including both arm abduction and elbow flexion.

### 3.2 Statistical testing

Tables 1, 2 show the results of the paired t-test run on the cumulative acceleration and smoothness for the five most representative features, as provided by the PCA histograms. These tables highlight the t- and p-values for both biomarkers performed on each of the extracted kinematics. Table 1 highlights that the smoothness of the maximum of both the left and right knee flexion during a squat has a statistically significant difference between the pre- and post-treatment measurements. However, Table 2 highlights considerably more biomarkers with a statistically significant difference in the sit-to-stand; these include the smoothness of the maximum knee flexion on both sides, smoothness of the left arm abduction, and both the smoothness and cumulative acceleration of the maximum elbow flexion on both sides.

**Table 1:** Paired *t*-test results showing the *t* and *p* values, with the values in bold representing *p* < 0.05, for each of the most representative squat biomarkers.

Biomarker (Squat)	Cumulative acceleration t(p)	Smoothness t(p)
Right Knee Flexion (mean)	1.786 (0.090)	1.203 (0.244)
Right Knee Flexion (max)	0.126 (0.901)	2.324 **(0.031)**
Left Knee Flexion (mean)	1.907 (0.072)	1.196 (0.246)
Left Knee Flexion (max)	0.483 (0.635)	2.528 **(0.021)**
Right Knee Flexion (min)	0.203 (0.841)	1.385 (0.182)

The bold values highlight the statistically significant values (*p* < 0.05).

**Table 2:** Paired *t*-test results showing the *t* and *p* values, with the values in bold representing *p* < 0.05, for each of the most representative sit-to-stand biomarkers.

Biomarker (Sit-to-Stand)	Cumulative acceleration t(p)	Smoothness t(p)
Left Knee Flexion (max)	0.772 (0.450)	2.976 **(0.008)**
Right Knee Flexion (max)	0.558 (0.584)	2.401 **(0.027)**
Left Arm Abduction (mean)	0.675 (0.508)	3.586 **(0.002)**
Right Elbow Flexion (max)	2.451 **(0.024)**	3.592 **(0.002)**
Left Elbow Flexion (max)	2.364 **(0.029)**	2.604 **(0.017)**

The bold values highlight the statistically significant values (*p* < 0.05).

**Figure 6:**
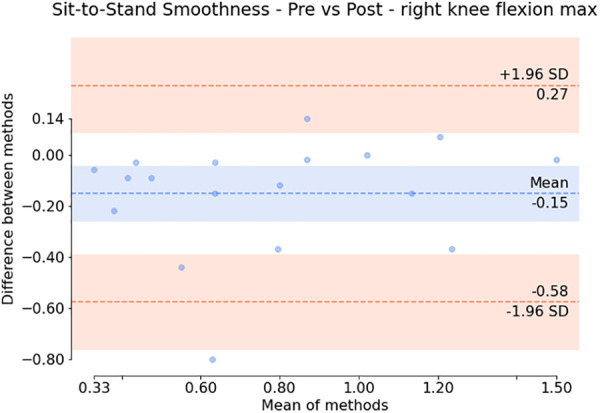
Bland Altman plot showing the difference against the mean for each patient (for the smoothness of the maximum right knee flexion) during the sit-to-stand action. The variation around the mean shows the apparent differences before and after the injection.

**Figure 7:**
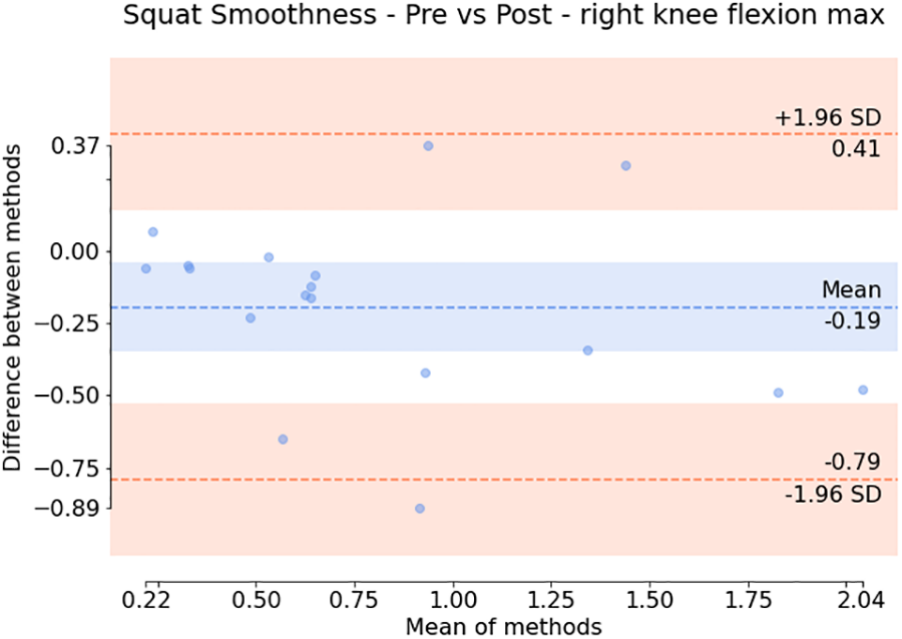
Bland Altman plot showing the difference against the mean for each patient (for the smoothness of the maximum right knee flexion) during the squat action. The variation around the mean shows the apparent differences before and after the injection.

Additionally, the subsequent Bland-Altman plots shown in Figures 7, 6 show that both the squat and sit-to-stand actions have a reasonable variability with most of the points falling within the two confidence intervals. The sit-to-stand action in Figure 6 shows a reduced variability with a consistent spread in the mean of differences as the mean of the methods increases. However, the squat action as shown in Figure 7 has an increase in the variability which can be seen by the larger increase in the mean of the differences as the mean of the methods increases.

### 3.3 Clinical trial results

**Figure 8:**
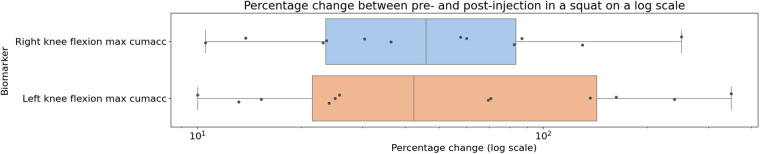
Box plot showing the median as well as the first and third quartile for percentage change for the entire clinical trial population from pre- to post-injection for the squat action projected on a logarithmic scale, showing the biomarkers identified to be statistically significant from the paired t-tests.

**Figure 9:**
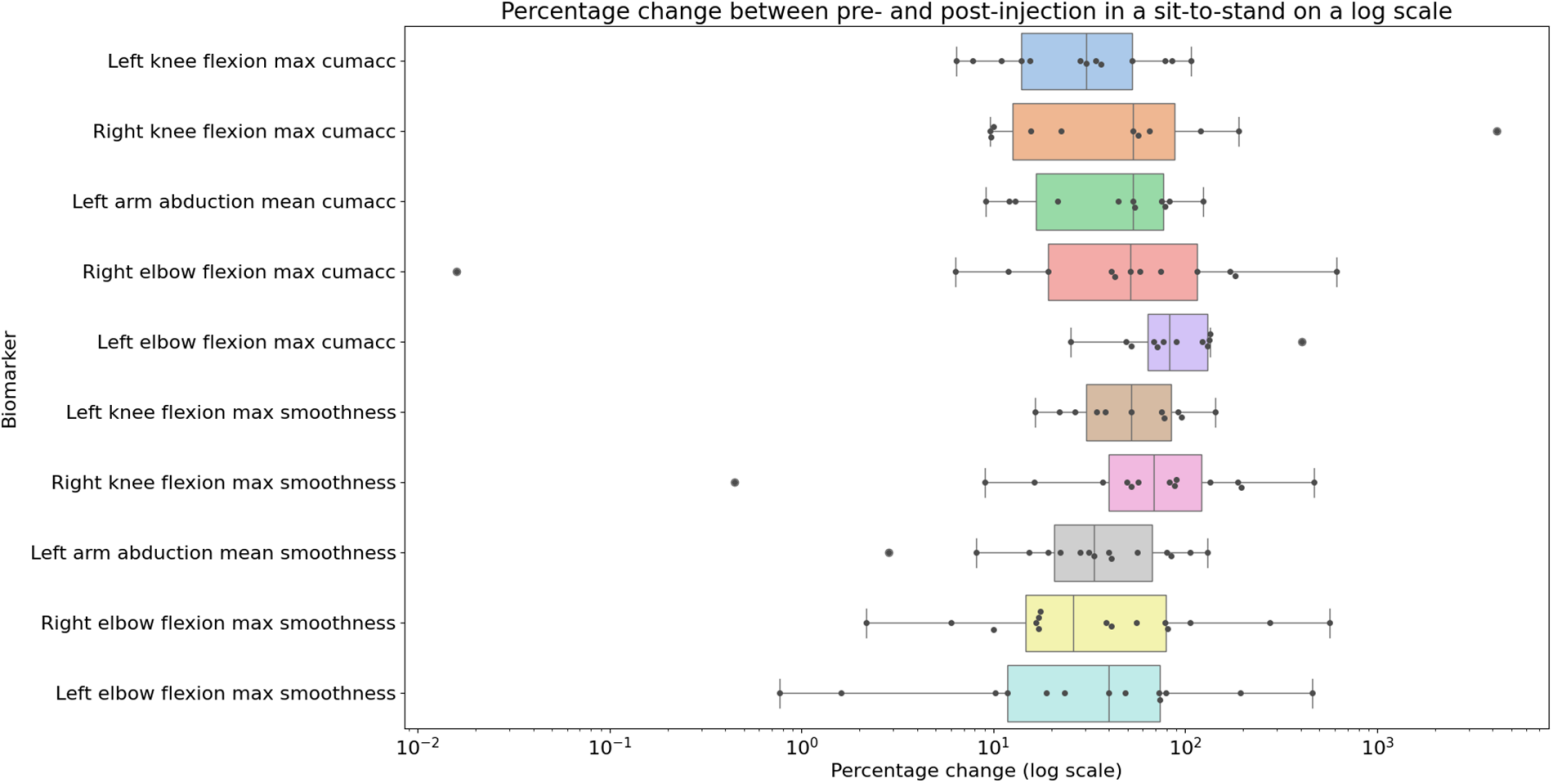
Box plot showing the median as well as the first and third quartile for percentage change for the entire clinical trial population from pre- to post-injection for the sit-to-stand action projected on a logarithmic scale, showing the biomarkers identified to be statistically significant from the paired t-tests.

The individual results from the clinical trial can be seen in Figures 8, 9, showing the change between the pre-and post-injection data for each of the statistically significant biomarkers identified in Tables 1, 2 for both the squat and sit-to-stand actions. These two figures show both the box and whisker plots to show the change for the entire group, as well as the scattered points to show the exact change for each individual. These results show that each of the biomarkers saw a median increase between the pre-and post-injection, however, there were some outliers in the data where the change was greater than expected or the value decreased after the treatment.

## 4 Discussion

In examining the PCA results depicted in Figures 4, 5, a pivotal revelation surfaces regarding the extraction of biomarkers from motion data. These histograms serve as a visual representation of the most significant features associated with each action. Notably, these identified features stand out as crucial candidates for biomarkers, showcasing their importance within the specific actions analysed. This finding holds profound implications, suggesting the potential utility of these biomarkers in diagnosing knee disorders and tracking disease or rehabilitation progression (35).

Paired statistical tests, differentiating pre-treatment and post-treatment, offer valuable insights into outcomes and methodological significance. Figures 6, 7 highlight crucial observations on the efficacy of the data collection technique. Results suggest the method establishes a sensitive coordinate system, detecting changes induced by treatment. Supported by participants receiving local anaesthetic, post-injection movements, devoid of knee pain, align more closely with physiological capabilities (36, 37). This nuanced understanding underscores the method’s importance in robustly assessing biomechanical changes post-treatment. However, Bland-Altman plots in Figures 6, 7 reveal areas for methodology improvement. While most participants show limited point spread, particularly in the squat action, outliers suggest selected biomarkers, though effective for many, may lack universal applicability. Consideration must be given to potential measurement error tied to treatment effectiveness variability rather than data collection or biomarker efficacy issues. Further investigation is crucial to pinpoint the source of this error, aligning with our overarching goal of precision in clinical biomechanics assessments. Furthermore, the effectiveness of these techniques in identifying biomarkers for knee disorders is evident in Figures 8, 9. Each biomarker displays a median percentage change increase, suggesting potential for monitoring movement capabilities. However, addressing anomalies in results, stemming from individualistic data, is crucial. Variations arise from the assumption of consistent movement patterns, not always holding true due to external factors like footwear, as demonstrated in prior research (38). Acknowledging and dissecting these intricacies are crucial steps in refining the application of these techniques for clinical biomechanics assessments, aligning with our overarching goals. An essential consideration in evaluating a technique’s diagnostic capabilities is the impact of measurement error. Nakano et al. report a mean absolute error in joint center location ranging from 20mm to 40mm, emphasising the challenge (39). Comparing with marker-based motion capture, a 50mm marker registration uncertainty results in a 7^∘^ peak joint angle variability (40). In contrast, inertial measurement units (IMUs) have up to 11.4^∘^ measurement error (41). This comparative analysis underscores the nuanced landscape of measurement errors, emphasising the need for a meticulous approach to enhance precision in clinical biomechanics assessments, a central objective of our study.

In addressing the challenge of measurement error, our approach is grounded in the assumption of non-differential measurement error. This decision is guided by our meticulous standardisation of variables for participants’ pre- and post-measurements. Every aspect, including the timing of measurements spaced 15-30 minutes apart on the same day, attire, lighting conditions, and the recording device utilising the same camera, is held consistent. Through this comprehensive standardisation, we maintain a uniform measurement error between both sets of measurements. While acknowledging potential effects on diagnostic accuracy, especially in comparisons between distinct groups, our primary focus centers on comparing the same individual before and after a specific treatment (42). This deliberate approach aligns seamlessly with our overarching goals, emphasising the crucial need for precision in clinical biomechanics assessments and contributing to the ongoing discourse on measurement error considerations.

The significance of these findings lies in the adaptability of the proposed methods for feature engineering across diverse applications. These techniques, showcased in a relatively small knee-based case study, carry implications that extend beyond the specific context. Their versatility allows seamless adaptation to various applications, addressing disorders not only in different body parts but also encompassing broader movement issues such as those associated with neurological disorders. It’s noteworthy that the actions performed in our study, though centered on the knee, can be tailored to suit the requirements of different applications. This adaptability underscores the broader potential of our techniques, aligning with our overarching goal of establishing a flexible and widely applicable framework for clinical biomechanics assessments.

A significant observation stems from the success of our feature extraction methodology, where each tested feature underwent initial extraction using PCA. Though further reduced for the paired t-test due to time series data constraints, these features provided descriptions based on both smoothness and cumulative acceleration from joint kinematics. Transforming these biomarker descriptions, as presented in Tables 1, 2, underscores their statistical significance. Beyond distinguishing actions from movement, these PCA-extracted biomarkers prove valuable in gauging treatment success (43, 44). This aligns with our broader goals, enhancing the potential for using motion-based biomarkers in clinical assessments and advancing our approach to clinical biomechanics. By synthesising the presented results, the described methods emerge as promising tools for clinical applications. These techniques successfully fulfill their objectives, providing a cost-effective solution for clinical biomechanics assessments. Importantly, their versatility extends beyond the confines of lower limb assessments, offering a wide range of potential applications. Beyond their diagnostic capabilities, this solution holds the potential to identify novel biomarkers associated with a diverse array of movement-debilitating conditions, including injuries, illnesses, and disorders. This multi-faceted approach aligns with our overarching goals of revolutionising clinical biomechanics, creating a scalable and adaptable framework with far-reaching implications for the field.

This study has played a crucial role in identifying the current limitations inherent in the described techniques. Notably, the absence of a controlled environment can introduce challenges, leading to occlusion and jitter problems, which necessitate attention in future research endeavors. Moreover, the limitations associated with the design of clinical trials come to the forefront. Establishing a truly representative sample proves challenging, and in this case, determining the prevalence of right-sided biomechanics raises intricate questions. It remains uncertain whether this prevalence is a result of the sample population being right-dominant or if patients experienced bilateral pain, thereby challenging the assumption of an equal distribution of pain in each knee. Recognising and addressing these limitations are vital steps in refining our methodologies, ensuring more accurate and comprehensive clinical biomechanics assessments aligned with our overarching goals.

In light of our current findings, we have identified promising avenues for future research and comparative studies, aimed at delving deeper into the capabilities of the proposed techniques. One such initiative involves a direct comparison between SMPL-based single RGB camera methods, MoCap, and IMUs. This comparative analysis seeks to elucidate the variability inherent in these different methods, providing valuable insights into their respective strengths and limitations. Additionally, we aim to extend the applicability of our motion-based biomarkers by introducing new disease pathologies to the dataset. This strategic expansion aims to assess the effectiveness of these biomarkers in distinguishing not only between diseases but also in discerning normal knee conditions. These future investigations align with our overarching goal of refining and broadening the scope of clinical biomechanics assessments, paving the way for advancements in remote monitoring and intervention strategies.

In conclusion, this study has not only demonstrated the effectiveness of utilising motion-based biomarkers for quantifying movement but has also established a robust foundation for conducting objective MSK analyses. The techniques presented offer a promising avenue for implementing a standardised method of MSK analysis, achievable with any standard camera, even a mobile phone. This accessibility opens doors for remote disease monitoring, enabling the early identification of pre-disease stages. By facilitating timely interventions, these methods have the potential to significantly alleviate the burden on the healthcare industry. In essence, our study contributes to the broader goal of revolutionising clinical biomechanics assessments, providing a low-cost solution with far-reaching applications beyond lower limb assessments, and paving the way for advancements in remote healthcare monitoring and intervention strategies.

## Data Availability

The raw data supporting the conclusions of this article will be made available by the authors, without undue reservation.
